# Nanoscale electron transport at the surface of a topological insulator

**DOI:** 10.1038/ncomms11381

**Published:** 2016-04-21

**Authors:** Sebastian Bauer, Christian A. Bobisch

**Affiliations:** 1 Faculty of Physics and Center for Nanointegration Duisburg-Essen (CENIDE), University of Duisburg-Essen, Lotharstrasse 1, 47057 Duisburg, Germany

## Abstract

The use of three-dimensional topological insulators for disruptive technologies critically depends on the dissipationless transport of electrons at the surface, because of the suppression of backscattering at defects. However, in real devices, defects are unavoidable and scattering at angles other than 180° is allowed for such materials. Until now, this has been studied indirectly by bulk measurements and by the analysis of the local density of states in close vicinity to defect sites. Here, we directly measure the nanoscale voltage drop caused by the scattering at step edges, which occurs if a lateral current flows along a three-dimensional topological insulator. The experiments were performed using scanning tunnelling potentiometry for thin Bi_2_Se_3_ films. So far, the observed voltage drops are small because of large contributions of the bulk to the electronic transport. However, for the use of ideal topological insulating thin films in devices, these contributions would play a significant role.

## Abstract (web summary)

Topological insulators offer lossless surface transport because of the suppression of conduction-electron backscattering from defect sites. Here, the authors show that the nanoscale voltage drops caused by such scattering can be directly measured using scanning tunnelling potentiometry.

## Graphical abstract

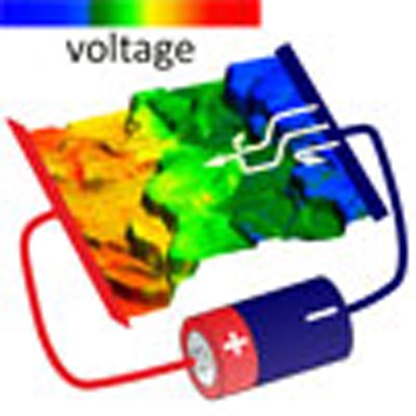

Both, the high conductivity and the spin polarization of the topological surface state imply the use of three-dimensional topological insulators (3D TIs)^1^,^2^ for spintronic devices (for example, for data processing). In addition, the low-energy dissipation due to the forbidden backscattering^3^,^4^,^5^,^6^ increases the efficiency and the lifetime of potential TI-based devices. Bi_2_Se_3_ is such a 3D TI with a bulk band gap of about 0.3 eV while the corresponding topological surface state is found to exhibit a nearly ideal Dirac cone^7^. The height of the band gap suppresses intrinsic conduction, so that Bi_2_Se_3_ is well suited for possible technical applications. In addition, the doping of Bi_2_Se_3_ can be tailored by low-level substitutions of Ca for Bi to adjust the Fermi level^8^. However, owing to unintentional doping by Se defects, the bulk of thin Bi_2_Se_3_ films is conductive. So far, the global sheet conductance of Bi_2_Se_3_ for different sample preparations has been studied on a macroscopic scale^9^,^10^,^11^,^12^. The microscopic scattering in (topological) surface states is mostly analysed by evaluating the lateral variation of the local density of states (LDOSs) using d*I*/d*V* measurements with a scanning tunnelling microscope (STM)^3^,^4^,^5^,^13^. Here, electron scattering at defects, for example, surface step edges is measured indirectly by analysing the lateral oscillations of the LDOS near the scattering centre. As this technique provides a high spatial resolution on the atomic scale^14^, the involved scattering channels are analysed with great spatial resolution^6^,^15^,^16^,^17^. This has also been supported by theoretical studies^15^,^16^,^18^,^19^, which proved that scattering at step edges on a topological surface is possible without violating the forbidden backscattering, decreasing the electron transport. Very recently, *ab-initio* calculations reveal the impact of Bi_2_Se_3_ step edges, that is, surface barriers on the electron transport^19^. For TI-based devices, local information on the electron transport through thin TI films on supporting substrates are relevant as processed device structures have reached dimensions of ∼14 nm already. For a 3D TI holding a surface current, the spatial variation of the electrochemical potential *μ*_ec_ at the surface carries direct and detailed information on the electron transport properties and the corresponding energy dissipation^20^. Datta shows that a local scatterer such as a step edge represents a one-dimensional Landauer-like resistive dipole, which results in a local drop of *μ*_ec_, that is, a local voltage drop at the defect site^20^. At the surface of a TI this has not been measured so far.

In the following, we use scanning tunnelling potentiometry (STP)^21^,^22^ to detect simultaneously *μ*_ec_ and the corresponding topography at the surface of the 3D TI Bi_2_Se_3_ with atomic-scale resolution. The analysis of *μ*_ec_ at the thin Bi_2_Se_3_ film surface yields a direct measure of current transport under realistic conditions, that is, a current of a few mA flows parallel to the surface analogue to real devices. We use thin films of Bi_2_Se_3_ on the technologically important substrate silicon with a film thickness of 14 QL, which ensures that the topological state is well established^23^, whereas the surface-to-bulk ratio of the film is still very high. This guarantees a maximum surface sensitivity. In particular, we find that the nanoscale electron transport in Bi_2_Se_3_ thin films is sensitive to surface step edges, manifested in local voltage drops at the step edge positions.

## Results

### Sample characterisation by macroscale transport measurements

**Figure 1:**
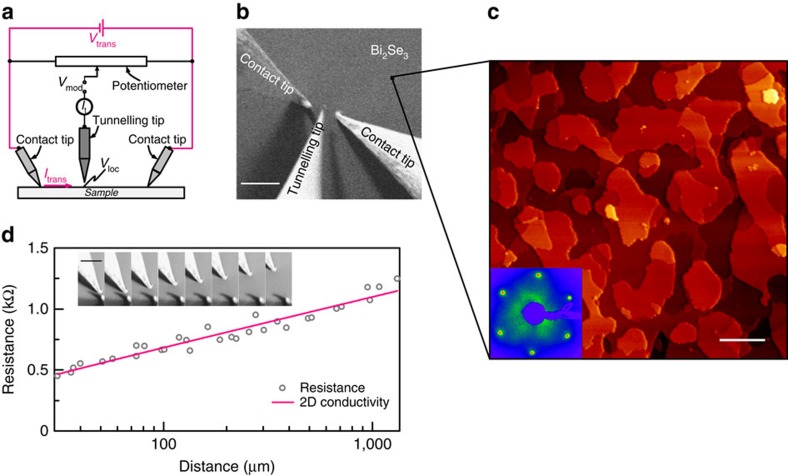
SEM, STM, LEED and sheet conductance of Bi_2_Se_3_ on Si(111). (**a**) Scheme of the STP experiment: a voltage *V*_trans_ leading to a transverse current *I*_trans_ is applied to the sample by two tips (red circuit). A potentiometer connects the tunnelling tip to the sample contacts in a Wheatstone bridge circuit. *V*_loc_ represents the local potential at the surface under the tunnelling tip. (**b**) SEM image of the contact geometry. Scale bar, 75 μm. Two Au tips contact the Bi_2_Se_3_ sample surface. A W tip is operated in tunnelling distance (tunnelling tip) and simultaneously maps the surface structure and the electrochemical potential. (**c**) STM image of the 14 QL Bi_2_Se_3_ film on Si(111) (*V*_t_=1 V, *I*_t_=12 pA, 650 × 650 nm^2^). Scale bar, 100 nm. The surface of the Bi_2_Se_3_ film is crystalline and flat. The inset shows the hexagonal LEED pattern of the Bi_2_Se_3_ film at 35 eV (lattice constant of 0.41±0.01 nm) exhibiting the high crystallinity of the film. (**d**) Distance-dependent resistance measurement of the Bi_2_Se_3_ film, leading to a sheet conductance of 1.8±0.1 mS. The inset shows SEM image snapshots of the measuring process. Scale bar, 75 μm.

**Figure 2:**
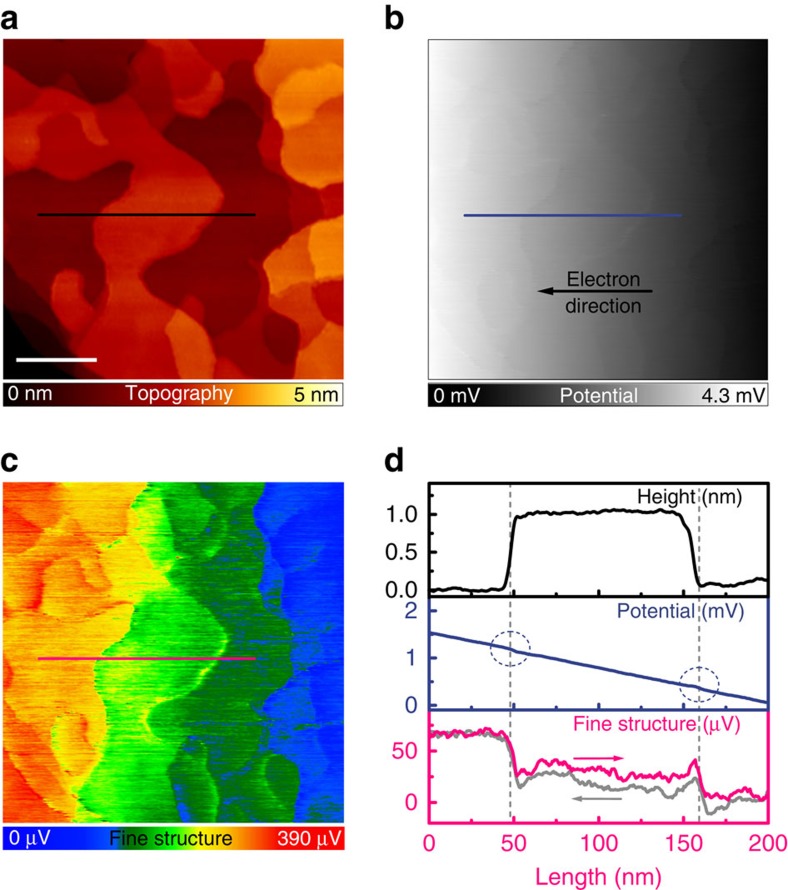
Results of the STP measurement. (*V*_mod_ =20 mVpp at 2.1 kHz, *I*_t_ = 12 pA, *V*_trans_=−1.3 V, *I*_trans_=−1.9 mA, 315 × 315 nm^2^). White scale bar, 75 nm. (**a**) Topography of the Bi_2_Se_3_ film. (**b**) The image of the potential for the corresponding surface area as in **a** mainly exhibits a constant gradient corresponding to the electric field induced by the contacting tips. (**c**) Corresponding ‘fine structure' of the potential (gradient subtracted). The colour coding helps to identify rather sharp drops of the potential at surface step edges. (**d**) Averaged line profiles (about 7 scan lines) across a Bi_2_Se_3_ island for the same position as marked in **a**,**b** and **c**. The plot of the fine structure includes the profile for the backward scan direction (light grey). The scan direction is indicated by arrows.

A sketch of the experimental setup and a SEM image of the contact geometry are shown in Fig. 1a,b. For further experimental details see the Methods. Figure 1c shows a typical STM image of the thin Bi_2_Se_3_ film. It reveals a layered structure with a step height for 1 QL of 1.01±0.07 nm (see also Fig. 2d) in agreement with other published data for Bi_2_Se_3_ (ref. 24). The inset in Fig. 1c shows the corresponding sharp and hexagonal low-energy electron diffraction (LEED) pattern indicating a high quality of the epitaxial film with a lateral lattice constant of 0.41±0.01 nm. Using a multi probe STM, we evaluate the macroscopic conductance of the sample *in situ*: Two tips contact the Bi_2_Se_3_ surface for different tip distances and the resistance between the tips is measured (inset Fig. 1d). Fitting of the data yields a (macroscopic) sheet conductance *G*_macro_ of the film of 1.8±0.1 mS (Fig. 1d), which agrees to the findings by other groups (for example, 1.3 mS for 10 QL Bi_2_Se_3_ on sapphire (0001), extracted from the diagram in ref. 25). This is discussed in detail in Supplementary Note 1 (see also Supplementary Fig. 1).

### Nanoscale transport measurements by STP

Figure 2 shows our experimental STP results, that is, an STM topography (Fig. 2a) and a simultaneously acquired map of the electrochemical potential (for short now called potential; Fig. 2b). Here, a lateral current of 1.9 mA is flowing through the sample from right to left (direction of the electrons). The potential mainly exhibits a constant gradient along this direction (Fig. 2c and line profile in Fig. 2e), which corresponds to an electric field *E* of 72±4 V cm^−1^. This constant gradient may result from phonon scattering as the dominant process within the Bi_2_Se_2_ film, which is not forbidden by the topological phase^26^,^27^.

For the given geometry, the average local current density *j* in the middle between the contacting tips can be estimated from the total transverse current *I*_trans_ by^28^


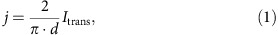


where *d* is the distance between the contact tips (for details see Supplementary Note 2 and Supplementary Fig. 2). Given the transverse current of *I*_trans_=1.9 mA and the distance between the contacting tips of *d*=80±20 μm (see Fig. 1b for details), the average local current density is *j*=0.15±0.03 A cm^−1^. Using Ohm's law, we calculate the local (microscopic) sheet conductance *G*_micro_ of the film: *G*_micro_=*j*/*E*=2.1±0.6 mS, which is close to the global film conductance of *G*_macro_*=*1.8±0.1 mS as determined from the resistance measurement at macroscopic distances (see Fig. 1d for details). This implies a rather homogeneous sheet conductance of the Bi_2_Se_3_ film. The sheet conductance *G* of a film is defined as the sum of the film's bulk conductivity *σ*_b_ times the film thickness *d* and the surface contribution *σ*_s_:


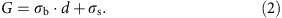


From literature^9^,^10^,^12^, we know that the surface conductivity of high-quality Bi_2_Se_3_ films is in the range of 0.4–0.8 mS. Thus, the dominating part (60–80%) for the microscopic conductance of our Bi_2_Se_3_ film is the bulk conductivity. This is plausible since during the film growth the Se excess was not very high (around 50% more Se atoms than necessary) and Se vacancies are incorporated into the film leading to an n-doping^8^,^9^. In consequence, around 30% of the current density flows through the surface state.

To analyse the local potential in detail, we subtract the macroscopic gradient from the measured potential and an additional ‘fine structure' in the potential becomes visible (Fig. 2c and line profile in Fig. 2d). This ‘fine structure' exhibits small voltage drops in the order of 20–30 μV, which are directly correlated to step edges in the topography (see Fig. 2a,b for details). To emphasise this, Fig. 2d shows line profiles across the same region for the topographic image, the potential image and the ‘fine structure'. In addition, we show the line profile for the ‘fine structure' for both, forward and backward scan direction. As the potential for both scan directions agree well, we can rule out a measurement artefact due to a non-ideal operating feedback loop for the tunnelling current.

### Excluding tip induced artefacts in the STP measurements

However, a drop in the measured potential may be caused by the geometry of the tip (that is, a double tip as discussed, for example, by ref. 21): In this case, the tunnelling junction is laterally displaced, leading to a voltage drop Δ*V* of the potential given by *E·d* with the electric field *E* and the distance *d* for the double tip. For the given values of *E* and Δ*V*, the distance would be ∼7 nm. Hence, the deviation of the constant gradient of the potential should be limited to a range of ∼7 nm. However, we find that the change of the potential at a step edge remains for distances in the order of 100 nm or more. This has been further corroborated by a numerical simulation taking care of different tip geometries and the measured topography. Here, it is worth noting, that the measured STP signals at the Bi_2_Se_3_ step edges are very small, reaching the limits of the STP measurement. Similar signals observed for the graphene surface (see ref. 28 for details) and the Si(111)-(√3 × √3)R30°-Ag surface^29^ are a magnitude higher (mV scale) than in our case (μV scale). Therefore, great care was taken to exclude artefacts. For a detailed discussion, see the Supplementary Note 3 inclusive Supplementary Figs 3 and 4.

Such a sharp voltage drop at a surface step edge is only observed in STP, if the underlying scattering event is located extremely close to the surface (M. Wenderoth and R. Möller, personal communication). Thus, the observed small voltage drops at surface step edges of Bi_2_Se_3_ clearly reveal that significant surface contributions are measured by our STP experiment and we conclude that a significant charge transport is mediated through the TI surface at room temperature.

### Detailed STP analysis at a 1 QL step

**Figure 3:**
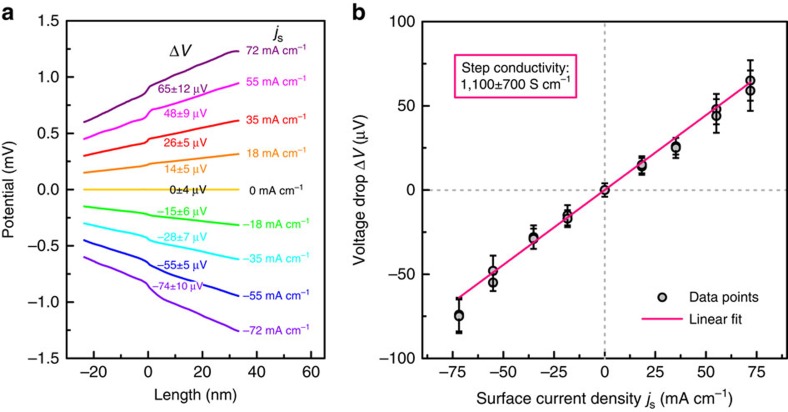
Step conductivity of a 1 QL step. (**a**) Averaged line profile of the potential in the vicinity of a 1-QL step of Bi_2_Se_3_ for different surface current densities *j*_s_ from −72 mA cm^−1^ to 72 mA cm^−1^. For clarity, the line profiles are vertically offset. The value of the voltage drop Δ*V* at the step edge is indicated. (**b**) Voltage drop at a 1-QL step as a function of surface current density. The linear fit yields the conductivity of a 1-QL step to 1,100±700 S cm^−1^. The individual error bars of the voltage drops represent the maximal error of each measured voltage drop as estimated from the noise level.

To analyse the voltage drop at a 1 QL step in more detail, Fig. 3a shows averaged line profiles of the potential across a 1 QL Bi_2_Se_3_ step for different surface current densities. The values of the observed voltage drops at the step edge are stated at each line profile. To improve the signal to noise ratio, we average the data for several scan lines (see also the Supplementary Note 4 and Supplementary Figs 5 and 6). By evaluating the voltage drop at the single QL step edge as a function of the surface current density (*j*_s_=−70 mA cm^−1^…+70 mA cm^−1^, Fig. 3b) we can deduce the conductivity at a QL step edge. As expected, for the low surface-current densities, the amplitude of the voltage drop scales linearly as a function of the surface current density. Now we compare an average local voltage drop of 30 μV with the observed gradient for a length of 100 nm, which is equivalent to a voltage difference of 720 μV. Using the fact that 30% of the current density flows through the surface state^9^,^10^,^12^, we estimate a voltage difference for a nominal length of 100 nm caused by phonon scattering to 210 μV for a pure surface conduction. Thus, the voltage drop at the step edge accounts to 15% for a length of 100 nm, which is a significant contribution to the surface state conductivity and will be even higher for an increased step edge density. Similar to the evaluation of the step conductivity induced by a substrate step of graphene on SiC in ref. 28, we determine the step conductivity of a 1 QL Bi_2_Se_3_ step from the slope of the fit to a numerical value of *σ*_step_=*j*_s_/Δ*V*=1,100±700 S cm^−1^ (Fig. 3b). The main factors for the uncertainties for the step conductivity are the measured distances between the two contact tips (25%) and the estimation of the portion of transvers current, which flows through the surface state (33%). Other error sources like the uncertainties in the measurement of the transverse current and in the measurements of the voltage drops are less important (some %). This leads to a total relative error of the step conductivity of ∼60%. From other STP data on Bi_2_Se_3_ (see Supplementary Note 5 and Supplementary Fig. 7), we can also make a rough estimation of the step conductivity of a 3 QL step to a numerical value of about 400 S cm^−1^. The impact of surface step edges to the lateral film conductance was also verified by macroscopic 4 point probe measurements. Here, we used a Bi_2_Se_3_ film with an anisotropic step distribution and find that the conductance of the film is also anisotropic. The resulting evaluation of the step edge conductivity from those macroscopic measurements yields a value of 1,000 S cm^−1^ for the step conductivity of the Bi_2_S_3_ steps in great agreement with the STP measurements. For more details, see the Supplementary Note 6 and Supplementary Figs 8–10.

## Discussion

The observed voltage drops demonstrate that the step edges are nano-resistances, which scatter the conduction electrons locally. This scattering process can be described as a reflection and transmission of incoming electron waves at the step edge, which does not contradict to the properties of a TI. Very recent *ab-initio* calculations by Narayan *et al*.^19^ show that Bi_2_Se_3_ step edges are barriers for the electron transport, which is consistent with our STP results. The direct backscattering (180°) is still forbidden, but other scattering channels (angles) are possible as discussed by Biswas and Balatzky^15^. This was already observed indirectly by d*I*/d*V* imaging, for example, on Bi_1-*x*_Sb_*x*_ by Roushan *et al*.^4^, on the Bi_2_Te_3_ surface by Alpichshev *et al*.^5^ and on the Bi_2_Se_3_ surface by Wang *et al*.^6^ Depending on the electron energy, the phase and the spin texture, the scattered electron waves interfere at the step edge with the incoming or transmitted electron waves resulting in oscillations of the LDOS near the step edge^14^,^16^, which diminish faster (power of −3/2) than for trivial two dimensional electron gas systems (power of −1/2) (ref. 6). Although direct backscattering for the surface state is not allowed, other non-180° scattering channels are available at the step edge, which limit the resulting conductivity of the Bi_2_Se_3_ surface. Here, it is worth noting that we cannot clearly distinguish if the scattering happens exclusively within the TI's surface state or if bulk states^30^ also participate in the scattering process. Epitaxial Bi_2_Se_3_ films are known to exhibit a n-doping because of Se vacancies, leading to a shift of the Fermi energy towards the valence band. At a high doping level, this would lead to an increased involvement of the bulk states in the scattering process. In realistic thin-film devices, such effects are also of technological relevance. Overall, our experimental results reveal that electron scattering also affects the local potential near a surface step edge of the 3D TI Bi_2_Se_3_ if a current is flowing through the surface. This is the first direct evidence for the relation between LDOS oscillations and local voltage drops at defect sites on TI surfaces.

Table 1 gives an overview on the step conductivities including other low-dimensional surface systems. The step conductivity of a 1-QL Bi_2_Se_3_ step (height of ca 1 nm) is similar to a SiC substrate step for a graphene layer^28^ (height of 0.5 nm). If we compare steps of the same step height (graphene on a SiC double step versus 1 QL Bi_2_Se_3_ step), the step conductivity of Bi_2_Se_3_ appears enhanced. This means that the impact of the step edge scattering in the case of Bi_2_Se_3_ thin films is reduced as compared with a substrate-supported graphene sheet. In comparison to, for example, a single step of the Si(111)-(√3 × √3)R30°-Ag (ref. 29) surface (height of 0.3 nm), the step conductivity of a 1-QL Bi_2_Se_3_ step appears 35 times higher. This implies that the overlap of the surface states on the upper and the lower terrace at the Bi_2_Se_3_ step edge is quite high. This is reasonable as the penetration depth of the surface state extents about 3 QL (ref. 23).

In conclusion, voltage drops at step edges of the Bi_2_Se_3_ surface reveal elementary contribution to the resistivity of TI surfaces. This proves that despite the topological protection the surface morphology plays a critical role for the electron transport, for example, in prospective thin TI film-based devices. For an ideal TI without any bulk contributions to the conductivity, the sum of the nano-resistances at step edges will limit the macroscopic surface conductivity. Our approach can also be applied to analyse scattering of conduction electrons at other defects structures such as domain boundaries, non-magnetic and magnetic adsorbates. With this knowledge, it should be possible to tune the electron transport of a TI surface on a local scale for designing smaller and more complex nanoscale device structures.

## Methods

### Sample preparation of Bi_2_Se_3_ thin films

For the preparation of Bi_2_Se_3_ films, we follow the recipe of Zhang *et al*.^24^ and Vyshnepolsky *et al*.^31^:

Bi (purity of 99.997% by Mateck) and Se (purity of 99.999% by Mateck) are co-evaporated with a ration Bi:Se of 1:2.25 (excess of Se) onto a Si(111)-(√3 × √3)-Bi substrate at room temperature^24^. The amount of deposited material is monitored by a quartz micro balance. The geometrical structure for the different steps of preparation is checked by LEED. A Si wafer with a low n-doping (phosphorus, conductivity of 7.7 mS cm^−1^) and a miscut of 0.5° (ca 30 Si-steps μm^−1^) was used. Before the deposition of Bi_2_Se_3_, the wafer was flashed to 1,500 K and slowly cooled to induce the 7 × 7 reconstruction. The equivalent of 10 ML of bismuth was deposited at a sample temperature of 300 K followed by heating to 720 K to prepare the Bi-(√3 × √3)-reconstruction. We grew a Bi_2_Se_3_ film with a nominal film thickness of ca 14 nm (about 14 QL), which is known to exhibit the topological phase^23^. Finally, the sample is annealed at 530 K for 2 h to ensure a flat and smooth film morphology^31^.

### Scanning tunnelling potentiometry

We use STP (Fig. 1a), which was first introduced by Muralt and Pohl^21^ in 1986. This is a STM-related technique, which allows us to measure the local electrochemical potential *μ*_ec_ and the local topography of the sample with atomic resolution simultaneously. In brief, two tips contact the sample and apply a voltage *V*_trans_ leading to a transverse current *I*_trans_ through the surface. A third tip is brought into tunnelling distance in the area between both contact tips. A potentiometer connects the contact tips and the tunnelling tip in a Wheatstone bridge circuit. If the bridge is balanced, the average DC tunnelling current *I*_t_ vanishes (〈*I*_t_〉=0) and the voltage at the tip matches the local potential *V*_loc_ at the position of the tunnelling tip. During scanning, the bridge is automatically readjusted for each position of the tunnelling tip and the voltage at the tip is recorded (see Bannani *et al*.^22^ for details). In addition, a small alternating bias *V*_mod_ is applied to the tunnelling junction resulting in an AC tunnelling current, which is used to control the distance between tip and sample. Thus, topographic STM imaging and potential mapping are simultaneously provided by STP. The geometry of the three tips and the sample surface monitored by SEM is shown in Fig. 1b.

## Additional information

**How to cite this article:** Bauer, S. and Bobisch, C. A. Nanoscale electron transport at the surface of a topological insulator. *Nat. Commun.* 7:11381 doi: 10.1038/ncomms11381 (2016).

## Supplementary Material

Supplementary Information Supplementary Figures 1-10, Supplementary Notes 1-6 and Supplementary References

## Figures and Tables

**Table 1:** Step conductivity *σ*_step_ of different 2D systems measured by STP*.

**System**	***σ***_**step**_ **(S cm**^−1^**)**
Si(111)-(√3 × √3)R30°-Ag (Si step)^29^	32±5
Si(111)-(√3 × √3)R30°-Ag (Si multiple step)^29^	7±3
Graphene on SiC (monolayer on a SiC step)^28^	1,400±500
Graphene on SiC (monolayer on a SiC double step)^28^	700±200
Graphene on SiC (monolayer on a SiC triple step)^28^	400±100
Bi_2_Se_3_ (1 QL step)	1,100±700
Bi_2_Se_3_ (3 QL step)	ca 400

2D, two dimension; STP, scanning tunnelling potentiometry.

^*^The step conductivities are measured perpendicular to the step edge.
