# Supplementary material for: Nanoscale electron transport at the surface of a topological insulator
Source: Nat Commun. 2016 Apr 21;7:11381. doi: 10.1038/ncomms11381 (PMC4844676; doi:10.1038/ncomms11381)
Supplement: Supplementary Information — Supplementary Figures 1-10, Supplementary Notes 1-6 and Supplementary References [file ncomms11381-s1.pdf]

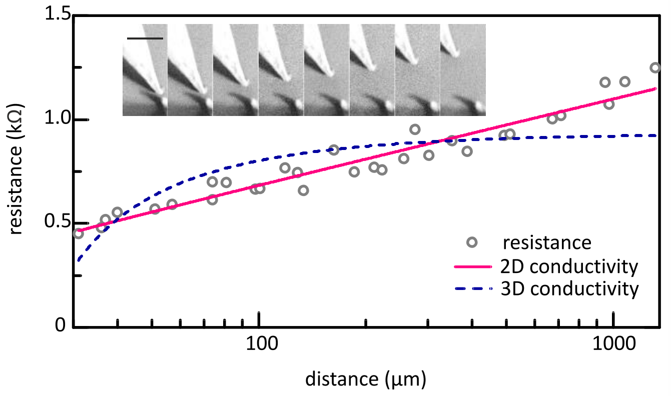


**Supplementary Figure 1 | Measurement of the sheet conductance.** Resistance as a function of probe spacing including 2D and 3D fits. The distance is plotted on a logarithmic scale. The inset shows corresponding SEM images for various probe spacing. Scale bar, 75 µm.

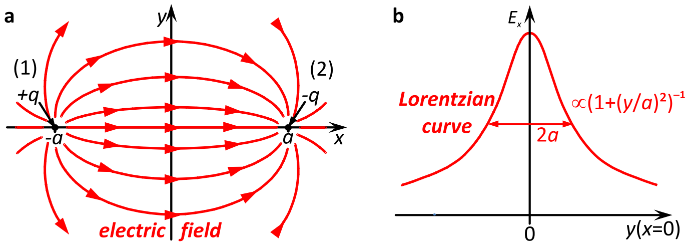


**Supplementary Figure 2 | Evaluation of the local current density *j*. a**, electric field distribution of two point charges (+$q$ and −$q$) on a homogeneous conducting surface. The distance between both point charges is $d$ = 2$a$. **b**, distribution of the electric field along the y-axis in (**a**) which can be described by a Lorentzian with a FWHM of 2$a$ (distance of the contact tips).

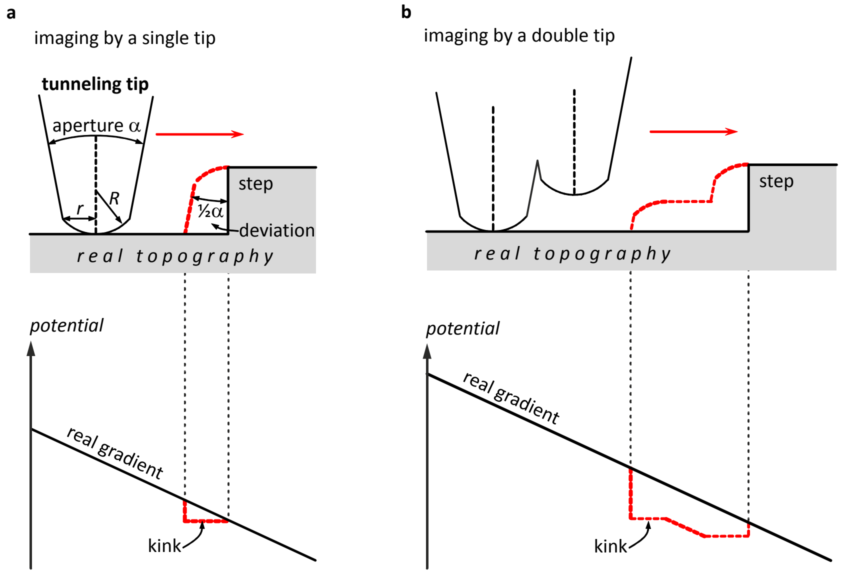


**Supplementary Figure 3 | Imaging process in STP. a**, Imaging process during the STP measurement by a single tunneling tip. A real tunneling has a certain width. All step edges of a surface appear expanded in the topography to the lower side of the step edge. If close enough, the expansion follows the potential of the upper terrace (the potential of the step edge). Therefore, kinks in the potential profile appears at step edges although in reality an e.g. linear gradient of the potential exists. **b**, Imaging process by a double tip.

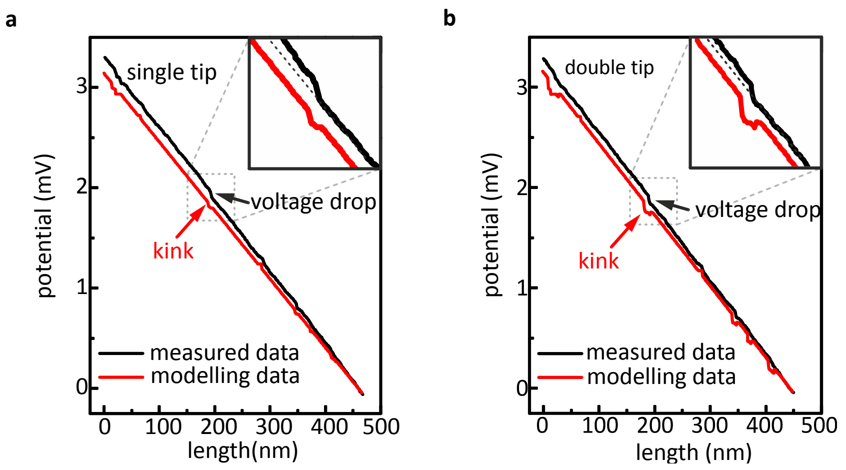


**Supplementary Figure 4 | Comparison of a simulation and the measured data of the potential.** The influence of the tip geometry to a linear gradient is compared to the measured gradient (black): **a**, single tip imaging; the voltage drops residing at a constant offset of the potential after the drop (black) cannot evolve from an imaging artefact by the single tip. **b**, double tip measurement; although the simulated kink in the potential is larger, the measured data cannot be modelled by double tip imaging.

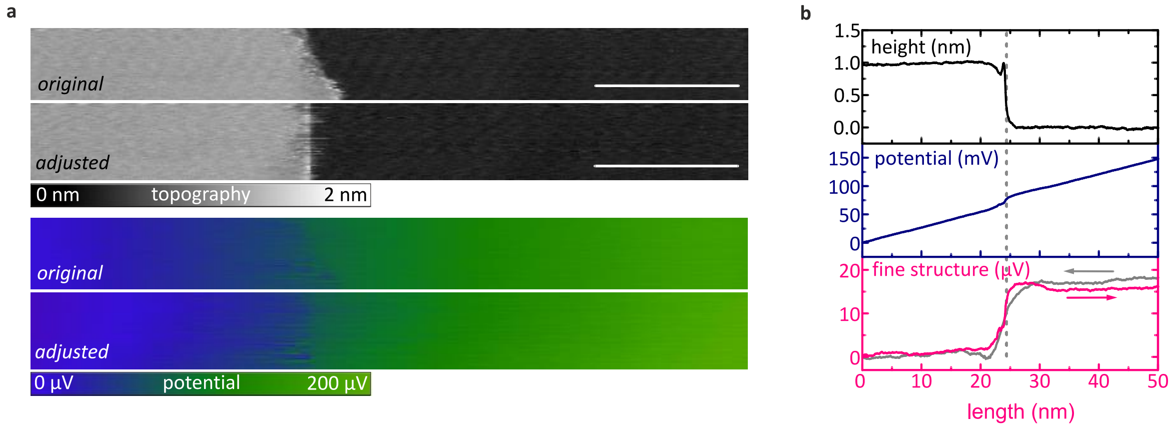


**Supplementary Figure 5 | Line adjustment of a 1 QL step edge. a**, Comparison of the original and adjusted STP images ($V_{mod}$ = 20 mVpp at 2.1 kHz, $I_t$ = 12 pA, $V_{trans}$ = 0.6 V, $I_{trans}$ = 0.8 mA, 67.4×6.7 nm² (scale bar, 13 nm) for the original data and 62.2×6.7 nm² (scale bar, 12 nm) for the adjusted data). **b**, corresponding line profiles which are averaged over all lines (average over 100 lines). In addition, also the line profile of the fine structure is shown. The voltage drop in the potential is directly correlated to the edge of the 1 QL step in the topography.

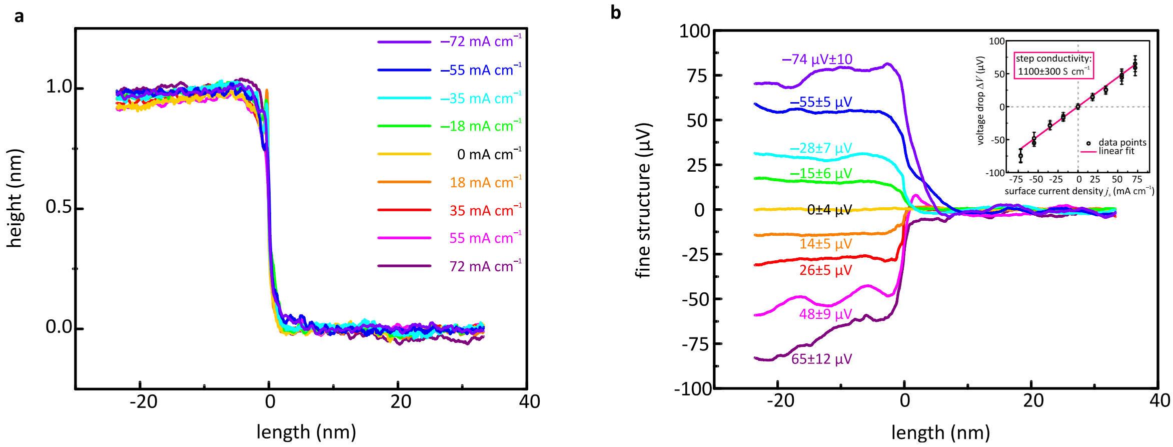


**Supplementary Figure 6 | Supplementary data to the profiles of the potential in Figure 3a in the manuscript. a**, Profiles of the topography at a $Bi_2Se_3$ corresponding to the profiles of the potential in Figure 3a. **b**, corresponding profiles of the fine structure including the value of each voltage drop (see (**a**) for the corresponding current densities). In good approximation, the voltage drop increasing almost linear as a function of the surface current density (see inset in **b**).

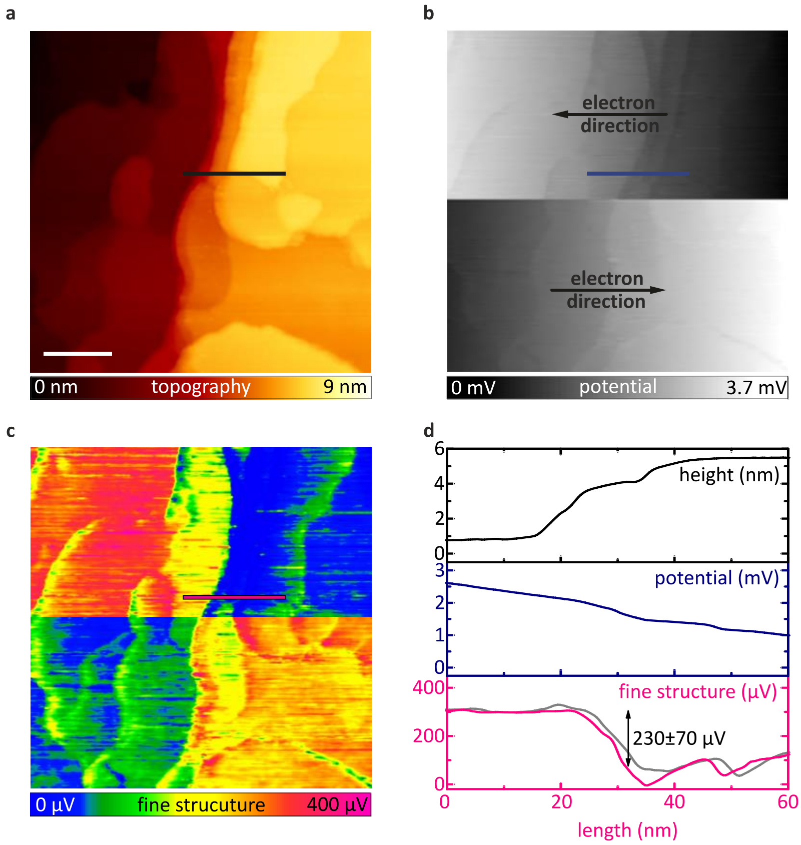


**Supplementary Figure 7 | STP measurement for a step bunch of $Bi_2Se_3$.** ($V_{mod}$ = 20 mVpp at 2.1 kHz, $I_t$ = 12 pA, $V_{trans}$ = ±2.1 V, $I_{trans}$ = ±3.3 mA, 250×250 nm²). **a**, Topography of the $Bi_2Se_3$ film. White scale bar, 50 nm **b**, Image of the potential of the corresponding surface area as in (**a**). A voltage drop at the 3 QL step is observed. **c**, Corresponding “fine structure” of the potential (gradient subtracted). **d**, Averaged line profiles (about 4 scan lines) corresponding to the position as marked in (**a**), (**b**), and (**c**). The plot of the fine structure includes the profile for the backward scan direction (light grey). The scan direction is indicated by arrows.

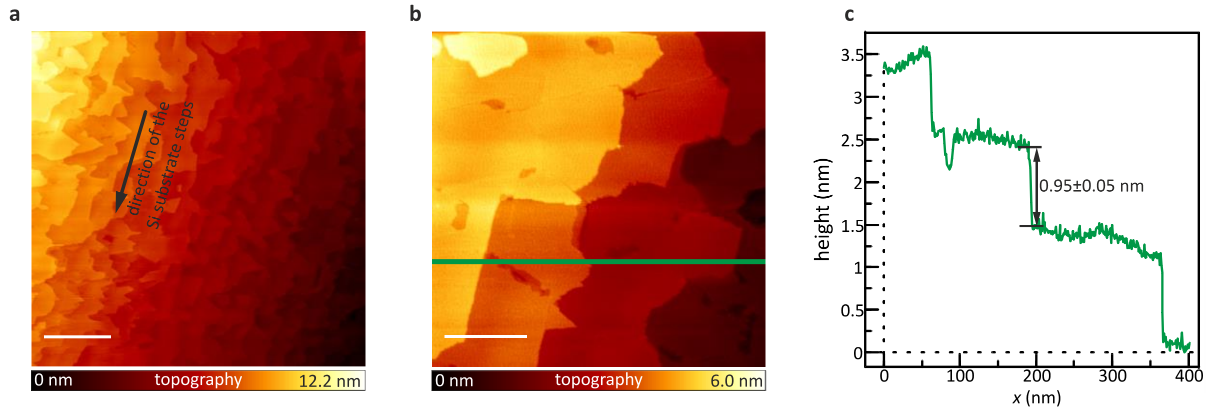


**Supplementary Figure 8 | topography of the stepped $Bi_2Se_3$ surface. a**, Large scale STM image of the $Bi_2Se_3$ surface ($V_t$ = 0.3 V, $I_t$ = 8 pA, 1.5×1.5 µm²). The $Bi_2Se_3$ surface structure is moderated by the underlying Si substrate and has evenly distributed steps which follow the direction of the Si steps. The step density is about $1\times10^5$ $cm^{-1}$. Scale bar, 300 nm. **b**, Close up of the $Bi_2Se_3$ film ($V_t$ = 0.3 V, $I_t$ = 8 pA, 400×400 nm²). Scale bar, 100 nm **c**, height profile of the indicated position in (**c**). The height of a $Bi_2Se_3$ layer is found at about 0.95 nm, corresponding to the theoretical value.

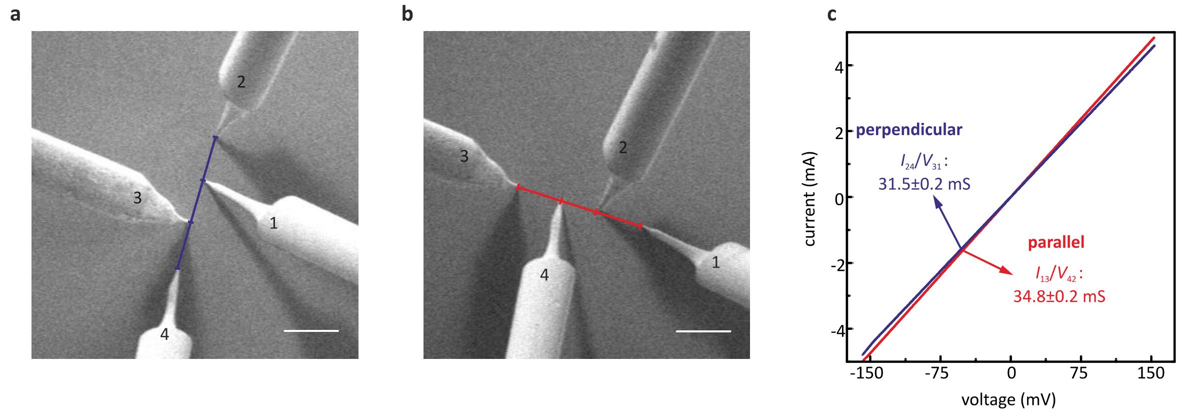


**Supplementary Figure 9 | Four point probe (4PP) measurements on the stepped $Bi_2Se_3$ surface (linear setup). a**, SEM image of the linear 4PP geometry perpendicular to the $Bi_2Se_3$ steps. Scale bar, 100 µm. **b**, SEM image of the 4PP geometry parallel to the $Bi_2Se_3$ steps. Scale bar, 100 µm. **c**, Recorded I-V- curve with the I-V-slope from a linear fit.

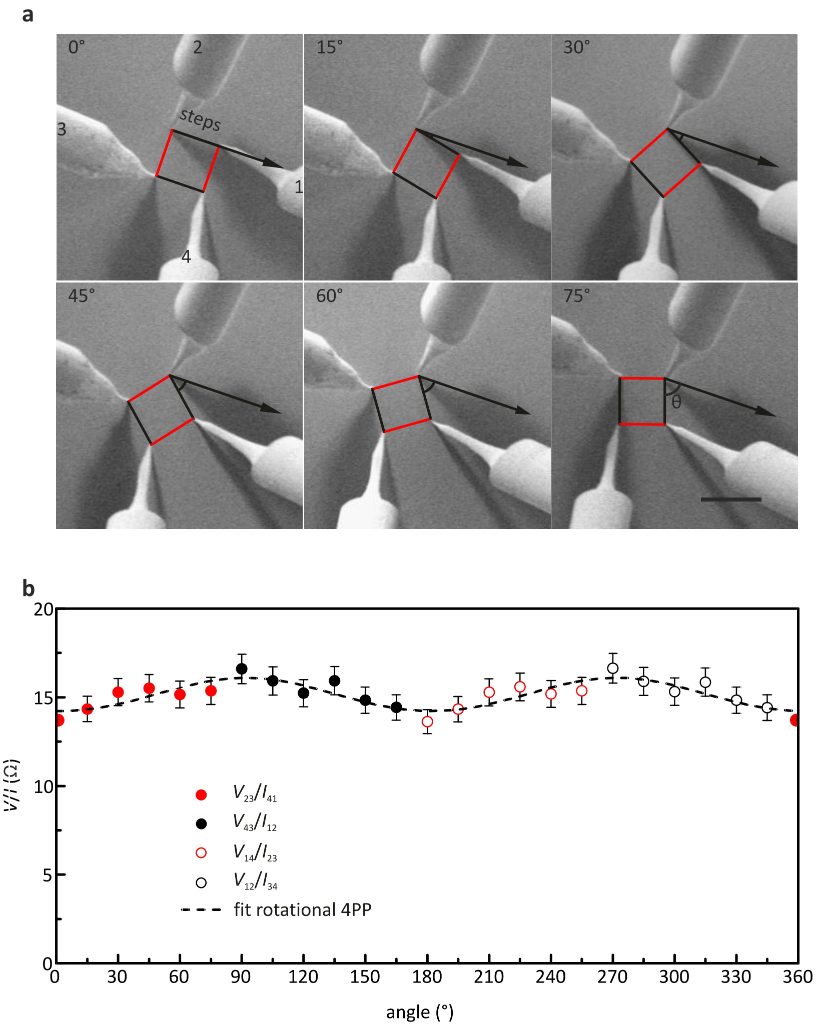


**Supplementary Figure 10 | Rotational square 4PP measurements on the stepped $Bi_2Se_3$ surface. a**, SEM images for various the rotational square 4PP position for different rotation angles θ. Scale bar 100 µm. **b**, 4PP resistance *V*/*I* vs. the rotation angle θ. The resistance shows a slightly, periodic dependence as a function of the angle of rotation. This indicates an anisotropic conductivity. The individual *V*/*I* error bars represent the maximum error (5%) caused by the fitting of the individual IV curves and the uncertainty of the tip position.

**Supplementary Note 1. Measurement of the Macroscopic Conductance of the Film**

We follow the method also used by Jachinsky et al.[1]: Two contact tips probe the sample for different probe spacing $d$ and the resistance $R$ between the tips is measured. Thereby we assume that the probe contact is almost ideal and the film conductivity almost isotropic. The resistance as a function of the probe spacing $R(d)$ differs for two cases, 2D ($\sigma_{2D}$) and 3D ($\sigma_{3D}$) conductivity[2]:

$$R_{2D}(d) = \frac{1}{\pi \cdot \sigma_{2D}} \cdot \ln\left(\frac{d}{r_c} - 1\right) \quad \text{and} \quad R_{3D}(d) = \frac{1}{\pi \cdot \sigma_{3D}} \cdot \left(\frac{1}{r_c} - \frac{1}{d - r_c}\right) \tag{1}$$

with $r_c$ as the average contact radius of the tips. $R_{2D}$ is valid, if the conducting sheet is thinner than the probe spacing (2D transport). $R_{3D}$ is valid, if the electron transport happens through the whole sample (3D transport) which in our case would also include the supporting silicon substrate. Note that $R_{3D}$ becomes constant for $d >> r_c$: $R_{3D} = (\pi \cdot \sigma_{3D} \cdot r_c)^{-1}$ = const. The comparison of both fits yields an estimation for the transport character (2D or 3D transport). In our experiment we varied the distance between the contact tips in the range from about 10 µm to 1000 µm while we observed the tip positions via a scanning electron microscope (see inset in Supplementary Figure 1). The results are shown in Supplementary Figure 1 including a 2D (red line) and 3D fit (blue intersected line).

Apparently, the 2D fit agrees a much better to the data. The 2D fit gives a value of $\sigma = 1.8 \pm 0.1$ mS and $r_c = 2.1 \pm 0.4$ µm and the 3D fit $\sigma = 280 \pm 30$ mS cm$^{-1}$ and $r_c = 12 \pm 1$ µm, respectively. If we compare the 3D fit results to the bulk conductivity of the Si wafer (7.7 mS cm$^{-1}$), the conductivity for the 3D fit yields a hundred times higher value than offered by the conductivity of the Si wafer. Also the fitting parameter of $r_c$ for the 3D case appears too large as compared to the estimated contact radius (Supplementary Figure 1). Therefore, we can deduce that the electron transport happens (almost) exclusively within the $Bi_2Se_3$ film. Since the film thickness $d$ (ca. 14 nm) is much lower than the probe spacing (10−1000 µm), the fitting parameter $\sigma_{2D}$ gives the conductance $G$ of the film. However, the surface contribution if the $Bi_2Se_3$ film is not be revealed.

**Supplementary Note 2. Evaluation of the Local Current Density *j* in Multiprobe STP Measurements**

In our STP measurements we use two contact tips to apply a transverse voltage $V_{\text{trans}}$ onto the surface, leading to a transverse current $I_{\text{trans}}$. To evaluate the local current density $j$, we use a static model similar to Ji et al.[3] to describe the distribution of the electric field $\boldsymbol{E}$ on the surface. We assume that the conductance $G$ of the film is homogeneous. The contact tips are modelled by two point charges with opposite parity and the same magnitude of $+q$ and $-q$ at $(-a,0)$ and $(a,0)$ on the surface (see Supplementary Figure 2a). The 2D electric field of a point charge is according to the divergence theorem $\mathbf{E} \propto \mathbf{r}/r^2$. Thus, the resulting electric field is a superposition of both point charges:

$$\mathbf{E}(x,y) = \begin{pmatrix} E_x \\ E_y \end{pmatrix} = \mathbf{E}_{+q} + \mathbf{E}_{-q} = C \cdot \left[ \begin{pmatrix} \frac{(x+a)}{(x+a)^2+y^2} \\ \frac{y}{(x+a)^2+y^2} \end{pmatrix} - \begin{pmatrix} \frac{(x-a)}{(x-a)^2+y^2} \\ \frac{y}{(x-a)^2+y^2} \end{pmatrix} \right] \tag{2}$$

where the constant C has the unit Volt. The electric field along the $y$ axis has the form of a Lorentzian (Supplementary Figure 2b),

$$\mathbf{E}(0,y) = 2C \cdot \begin{pmatrix} \frac{a}{a^2+y^2} \\ 0 \end{pmatrix} = E_0 \cdot \begin{pmatrix} \frac{1}{1+y^2/a^2} \\ 0 \end{pmatrix}, \tag{3}$$

with $E_0 = E(0,0)$. We assume that the transverse current flows through the surface without changing the electric field (quasistatic approximation). From Ohms law we know that $j = G \cdot E$. The integral of equation (3) over d$y$ multiplied with the conductance $G$ is equal to the total current $I_{\text{trans}}$ flowing perpendicular to the $y$ axis:

$$I_{trans} = G \int_{-\infty}^{+\infty} E(0,y)\,\mathrm{d}y = j_0 \int_{-\infty}^{+\infty} \frac{1}{1+(y/a)^2}\,\mathrm{d}y = j_0 \cdot \pi a, \qquad (4)$$

where $j_0 = j(0,0)$ is the current density at the origin in Supplementary Figure 2a. Equation 1 in the main manuscript is a rearrangement of equation 4. In our experiments the tunnelling tip recording the electrochemical potential is placed in the middle between the contact tips (corresponding to the position (0,0), see also Figure 1 in the main manuscript).

Thus, with the knowledge of the total transverse current $I_{\text{trans}}$ and the distance between both contact tips $2a$ we can calculate an estimated total current density $j_0$ for our STP measurements.

## Supplementary Note 3. Imaging the Potential by STP - Principle and Simulation

In literature, the influence of the tip geometry on the topography in STM images is often neglected. However, a clear picture of the imaging process is necessary for the interpretation of STP measurements and especially for the identification of artefacts[4]. If the tunnelling tip is laterally approaching the step edge (see Supplementary Figure 3a) the point of tunnelling (shortest distance between the surface and the tunnelling tip) on the surface changes from the terrace to the upper kink of the step edge. Simultaneously, the point of tunnelling at the tip changes from the front end to the side (apex) of the tip. In close vicinity to the step edge and during scanning across the step edge, the step edge serves as one tunnelling point while the tip is moved upwards across the step edge. Thus, during scanning the step edges appear broadened in the acquired STM image. This broadening has the shape of half the tip apex (see Supplementary Figure 3a). In other words: for the step edge position, the step edge serves as the “tunnelling tip” and “scans” the tip’s apex. If we now assume a constant gradient of the potential along the scanned surface, a little kink in the potential would be recorded in the STP measurement while the step edge is imaged. At the position where the geometry of the tunnelling gap changes, the potential on the upper terrace determines the imaged potential, which appears then shifted away from its real position at the step edge towards the position of the STM tip. Thus, a sharp transition of the potential would occur, copying the value of the potential of the upper terrace to the left hand side. Finally, the junction geometry on the upper terrace away from the step edge reflects the same geometry as before approaching the step edge, recovering the gradient of the potential.

We simulated the imaging process for our data to interpret the fine structure found in our STP data. Therefore, we analysed the broadening of the step edges to deduce the shape of the tip apex. To model the imaging process, the tip is assumed with a sphere like geometry at the front end with a curvature radius $R$ followed by a cone with an aperture $\alpha$ and a cone radius $r$ as depicted in Supplementary Figure 3a. Accordingly, we had to shrink the lateral size of the measured topography and afterwards simulated the tip imaging process. By subsequently changing the parameters of the tip apex and the shrinking factor until the simulated image reflects the measured image, we can extract the original topography. From this fitting procedure, we can assume an apex radius $r$ of 8 nm and an aperture of 140° and a curvature radius of 15 nm for our used STM tip.

With this tip, the influence on the potential is then simulated for the case of a constant gradient of the potential. Supplementary Figure 4a shows a comparison of the simulated potential (red) with the measured data (black) for a single tip imaging process. The simulated potential exhibits a small kink at the step position and recovers to the constant gradient soon after the step edge position. In contrast, the measured data (black) exhibits a drop in the potential at the step edge position and resides at a constant offset. Obviously, the measured voltage drop cannot be explained by the imaging process with the tunnelling tip.

Similarly, the potential for a double tip was also simulated (see Supplementary Figure 3b and 4b). Both apices of the double tip are set to the same parameters like the single tip. The lateral distance between the tips was chosen at 7.5 nm. Supplementary Figure 4b shows that in this case a broader kink at the step edge position is found whose extension reflects the lateral distance for the double tip. The corresponding measured data shows the drop of the potential with the constant offset. The evolving kink of the simulated potential reflects the difference of the potential corresponding to the distance of the double tip. If we again compare the measured and the simulated potential, we see that there exists a drop in the simulated data similar to the measured potential, but it does return to the constant gradient soon after. In contrast, the measured potential drop results in a constant offset.

In conclusion, for both possible tip artefacts we can exclude that the measured voltage drop in our data is caused by a single or double tip artefact. However, the exact shape of the transition of the potential at the step edge is not resolved.

#### Supplementary Note 4. Investigation of the Step Conductivity of a 1 QL Step

For the determination of the step conductivity of a 1 QL step edge, a stripe of the sample surface including a single quintuple step was analysed by STP (see Supplementary Figure 5a). The step edge in the topographic image was adjusted into a vertical line and the excess end of the scan lines were cut away. The potential was adjusted according to the line shifting and cutting in the topography. The average of all adjusted lines in vertical direction increases the signal-to-noise ratio and allows a better analysis of the voltage drop.

Supplementary Figure 6 shows the corresponding profiles of the topography and of the fine structure of the potential from Figure 3a in the main manuscript. In contrast to the data shown in the manuscript, here the constant gradient was subtracted from the data. The step edge is recorded at different surface current densities and the increase of the corresponding voltage drop as a function of the surface current density is nicely shown (Supplementary Figure 6b).

#### Supplementary Note 5. STP at a $Bi_2Se_3$ Step Bunch

Supplementary Figure 7 shows the STP measurement at a step bunch of the $Bi_2Se_3$ film. A voltage drop is also observed at the $Bi_2Se_3$ step bunch. The image includes a change of the polarity of the transverse current at the middle of the image. Obviously, the voltage drop and the gradient changes its sign if the polarity is changed. This proves that the voltage drop is induced by the gradient of the potential. The voltage drop at a 3 QL step has a value of $\Delta V$ = 230±70 µV. The larger error is caused by the reduced quality of the fine structure signal in the data. The transverse current was set to 3.3 mA. From equation 1 in the main manuscript we assume a surface current density is $j_s$ = 80 mA cm$^{-1}$. This leads to a step conductivity of about 400 S cm$^{-1}$ for a 3 QL step.

#### Supplementary Note 6. Additional Four Point Probe Measurements on a stepped $Bi_2Se_3$ Surface

We prepared another $Bi_2Se_3$ film on an equivalent Si(111) substrate (same wafer as for the other date with a miscut of 0.5° (single step density 3×10$^5$ cm$^{-1}$) and a conductivity of 7.7 mS cm$^{-1}$). The preparation was analogue compared to the first sample except the annealing procedure. Here, the sample was annealed to a higher temperature of 570 K. This procedure influences the occurrence of $Bi_2Se_3$ step edges. Supplementary Figure 8a shows a large scale STM image of the $Bi_2Se_3$ surface. The surface shows a regular step array. The steps follow the direction of the underlying Si(111) substrate steps as determined before $Bi_2Se_3$ growth. The $Bi_2Se_3$ step den-

sity is about $(1.1\pm0.1)\times10^5$ cm$^{-1}$ and appears three times smaller as compared to the density of the Si single steps density. Three Si steps have a height of about 0.93 nm, which is very close to the height of a quintuple layer (0.95 nm). Thus, at higher annealing temperature, the film relaxes and accommodates the step formation of the silicon substrate. In this sense, it is easier for the $Bi_2Se_3$ layers to grow in a regular stepped array on the Si(111) surface.

Since this regular step array is present over large portions of the $Bi_2Se_3$ film area and induces an anisotropic step edge distribution also mesoscopic transport measurements can give access to the scattering at $Bi_2Se_3$ step edges. The electric conductivity of the film was measured by a classical four point probe (4PP) measurement in linear geometry (see Supplementary Figure 9).

Four tips were arranged in a line (distance between the tips about 80 µm) and a current is applied through the outer tips and the corresponding voltage drop between the inner tips is measured. The *I*/*V*-slopes from the *I*-*V*-curves were measured for a transverse current parallel and perpendicular to the direction of $Bi_2Se_3$ step edges. The *I*-*V*-measurements yield a *I*/*V* value of 31.5±0.2 mS for the parallel case and 34.8±0.2 mS for the perpendicular case (see Supplementary Figure 9c).

The sheet resistance can be calculated from the *I*/*V*-slope by using the following equation:[5]

$$G = \frac{\ln(2)}{\pi}\frac{I}{V}. \tag{5}$$

Here, we obtain a sheet resistance of $G_{\text{parallel}} = 7.7\pm0.8$ mS und $G_{\text{vertical}} = 7.0\pm0.7$ mS. Thus, the surface conductivity seems to be anisotropic. However, one has to keep in mind that the linear 4PP yields the geometric mean $(G_{\|}\cdot G_{\perp})^{1/2}$ of the surface conductivity.[4] A correct information about the anisotropy of the film's conductivity cannot be given. To access the transport components in parallel ($G_{\|}$) and perpendicular ($G_{\perp}$) to the direction of the $Bi_2Se_3$ step edges, a 4PP measurement in a square geometry is used (see Supplementary Figure 10a).[5]

The current flows between tip 1 and 4 and the voltage is measured between tip 2 and 3. During the measurement, the position of the resulting contact square is rotated stepwise from 0° to 90 (see Supplementary Figure 10a). The pairs of current carrying tips and voltage measuring tips permutes cyclic for every rotational step. Therefore, we have access to the 4PP resistance between 0 and 360°. The angular dependence of the resistance *V*/*I* of the 4PP measurement is given by[5]

$$\frac{V}{I} = \frac{1}{2\pi\sqrt{G_{\|}\cdot G_{\perp}}}\times\ln\sqrt{\frac{(G_{\|}/G_{\perp}+1)^2 - 4\cos^2\theta\sin^2\theta\,(G_{\|}/G_{\perp}-1)^2}{(\sin^2\theta + G_{\|}/G_{\perp}\cdot\cos^2\theta)^2}} \tag{6}$$

where θ is the angle of rotation. Fitting of equation 6 to the data in Supplementary Figure 10b yields $G_{\|} = 7.60\pm0.06$ mS and $G_{\perp} = 6.98\pm0.06$ mS. Here, a clear dependence between the conductivity and the direction of the step edges is found. The relation $G_{\perp}\backslash G_{\|} = 1.09\pm0.02$ shows an anisotropy of (9±2)% Assuming that the step edges give additional contributions to the sheet resistance of the film, the conductivity of individual step edges can be computed by[6]

$$\sigma_{\text{step}} = n_{\text{step}}\frac{G_{\perp}\cdot G_{\|}}{G_{\|}-G_{\perp}}, \tag{7}$$

where $n_{\text{step}} = (1.1\pm0.1)\times10^5$ cm$^{-1}$ is the density of $Bi_2Se_3$ steps on the surface. Inserting all values gives a step conductivity of about 9400 S cm$^{-1}$. Here, we make the same assumptions as in the main manuscript. The scattering predominantly happens in the surface state. The essential contribution of the surface state to the total sheet conductance (about 7 mS) is assumed to about 10% (0.4 mS – 0.8 mS) which gives a value of about 1000 S cm$^{-1}$ for the step conductivity of the $Bi_2S_3$ steps. This value matches the value calculated from the STP data of the microscopic potentiometric measurement with reasonable agreement and verifies the corresponding findings.

## Supplementary References


1. Jaschinsky, P., Wensorra, J., Lepsa, M. I., Mysliveček, J., Voigtländer, B. Nanoscale charge transport measurements using a double-tip scanning tunneling microscope. *J. Appl. Phys.* **104**, 094307 (2008).
2. Hofmann, P., Wells, J. W. Surface-sensitive conductance measurements. *J. Phys. Condens. Matter* **21**, 013003 (2008).
3. Ji, S. H. *et al.* Atomic-scale transport in epitaxial graphene. *Nature Mat.* **11**, 114-119 (2011).
4. Pelz, J. P & Koch, R. H. Tip-related artifacts in scanning tunneling potentiometry. *Phys. Rev. B* **41**, 1212 (1990).
5. Kanagawa, T. *et al.* Anisotropy in Conductance of a Quasi-One-Dimensional Metallic Surface State Measured by a Square Micro-Four-Point Probe Method. *Phys. Rev. Lett.* **91**, 036805 (2003).
6. Matsuda, I. *et al.* Electrical Resistance of a Monatomic Step on a Crystal Surface. *Phys. Rev. Lett.* **93**, 236801 (2004).
